# Assessment of Muscle Stiffness Using the MyotonPro: Effects of Fatigue on Vastus Lateralis and Medialis Muscles

**DOI:** 10.3390/jpm14030301

**Published:** 2024-03-12

**Authors:** Jonathan Lettner, Lars Graventein, Hassan Tarek Hakam, Nikolai Ramadanov, Roland Becker, Robert Prill

**Affiliations:** 1 Center of Orthopaedics and Traumatology, University Hospital Brandenburg/Havel, Brandenburg Medical School, Hochstraße 29, 14770 Brandenburg an der Havel, Germany; jonathan.lettner@mhb-fontane.de (J.L.);; 2 Faculty of Health Science, Brandenburg Medical School, 14770 Brandenburg an der Havel, Germany

**Keywords:** MyotonPro, vastus lateralis muscle, vastus medialis muscle, maximal muscle fatigue, stiffness, frequency, reliability

## Abstract

Background: The investigation of soft tissue stiffness has garnered increasing interest due to its potential applications in detecting tissue conditions, monitoring therapy effects, and preventing sports injuries. This study utilized the MyotonPro as a reliable measurement device to assess muscle stiffness and muscle frequency in the vastus lateralis and medialis muscles of both the dominant and non-dominant legs. Methods: Sixteen young, healthy subjects (seven males and nine females, age 25 ± 3.46 years) participated in this study. To induce maximal muscle fatigue, the vastus lateralis and vastus medialis muscles were subjected to a 30 kg load using a single-leg leg press. Pre- and post-fatigue measurements were conducted by two testers on the dominant and non-dominant legs, respectively, employing the MyotonPro. Results: We revealed a significant increase in muscle stiffness after maximal muscle fatigue. Specifically, on the dominant side, the vastus lateralis exhibited a stiffness increase of 6.5%, while the vastus medialis showed a 6.3% increase. On the non-dominant side, the vastus lateralis demonstrated a 7.6% increase, and the vastus medialis exhibited a 6.7% increase in muscle stiffness. Furthermore, muscle frequency increased by 8.6% (vastus lateralis) and 13.5% (vastus medialis) on the dominant side and by 15.1% (vastus lateralis) and 6.3% (vastus medialis) on the non-dominant side. The reliability of the measurements varied, with Cronbach’s alpha values ranging from inadequate 0.49 to very good 0.88. Conclusion: This study affirms the efficacy of the MyotonPro as a measurement device for assessing muscle stiffness and establishes its reliability. The observed increase in muscle stiffness after maximal muscle fatigue, accompanied by changes in muscle frequency, underscores the device’s utility. However, further research is warranted to validate the reproducibility of these findings and explore additional facets of the muscular response to fatigue.

## 1. Introduction

In recent years, there has been a growing interest among researchers and clinicians working with patients and athletes in the field of soft tissue stiffness, which refers to the resistance of biological structures to external deformation [1,2,3,4,5]. This increasing interest is driven by preliminary findings suggesting the crucial role of soft tissue stiffness in detecting pathological tissue conditions, monitoring therapeutic or training effects, and preventing sports injuries. Soft tissue stiffness serves as a vital parameter, providing insights into the structural properties and function of both muscle and connective tissue.

An illustrative example of this phenomenon is the noticeable increase in stiffness observed among basketball players during the transition from adolescence to adulthood, highlighting the intricate interplay between age-related transitions and musculoskeletal characteristics [6]. Elevated levels of stiffness may serve as indicators of inflammatory processes, injury, or degenerative changes, while reduced stiffness may suggest diminished muscular function or a lack of tissue elasticity. Bas et al. established a significant association between the number of falls in older individuals and reduced muscle stiffness of the gastrocnemius muscle in their study [7].

Furthermore, Evangelidis et al. demonstrated that muscle fatigue significantly impacts the stiffness of the biceps femoris long-head muscle more than the semitendinosus and semimembranosus muscles, providing insight into the increased vulnerability to strain injuries within this muscle group [8]. Additionally, in the context of rehabilitation following Rotator Cuff tendinopathy, there is a manifestation of reduced tendon stiffness [9].

The potential of soft tissue stiffness as a diagnostic and monitoring tool underscores the need for comprehensive investigations. In this context, the present study employs the MyotonPro as a reliable measurement device to delve into the effects of maximal muscle fatigue on the stiffness and frequency of the vastus lateralis and medialis muscles. The objective is to contribute to the growing body of knowledge surrounding soft tissue mechanics and its applications in healthcare and sports science [10,11,12,13,14,15,16].

Several devices are available for evaluating and quantifying muscle stiffness, with the MyotonPro (Myoton AS, Tallinn, Estonia) chosen as the measurement instrument for the purposes of this study. The MyotonPro facilitates the assessment of various parameters, including stiffness, decrement, frequency, creep, and relaxation. However, for the scope of this research project, only Stiffness and Frequency were investigated, with the other parameters not being further considered.

Muscle stiffness, a key property explored in this study, is a significant characteristic that delineates the resistance of biological soft tissues to external deformation forces. It provides insights into how robustly a tissue responds to external forces and its ability to maintain its structural integrity [17] (pp. 216–266). Stiffness is governed by the structural attributes of the tissue, such as the arrangement and density of collagen fibers in connective tissue or the contractility of muscle fibers in muscle tissue. The greater the stiffness of a tissue, the more resistant it is to deformation, resulting in lower elasticity [17] (pp. 267–271). Reduced muscle stiffness and muscle tone lead to diminished postural control yet concurrently coincide with enhanced neuromuscular performance [18].

The MyotonPro device measures muscle stiffness using gentle mechanical stimulation. It is placed on the skin over the muscle to be examined and generates a short pulse that causes the tissue to vibrate.

Based on the return time and the damping of the vibration, MyotonPro calculates the stiffness of the tissue, which is expressed in Newton/meter (N/m). The MyotonPro calculates the stiffness as follows: $S=\frac{a_{\max\;\cdot \;m_{probe}}}{∆l}$. Muscle frequency is another important parameter that characterizes the properties of muscle tissue. It indicates how fast muscles are able to contract and relax, and it is also an indicator of the recruiting capacity. Muscle frequency is often considered a measure of the muscle’s responsiveness to electrical impulses and can provide clues to neuromuscular control and function. Frequency is calculated as follows: $F\equiv f_{max}$.

While the reliability [19] of the MyotonPro has been explored in various studies, particularly in neurology [20,21,22], its consideration within the realm of maximal muscle fatigue and the potential influence of these muscle property alterations on its reliability has yet to be investigated [20,23,24,25,26]. This proves particularly intriguing, as long-term low-intensity running training fails to induce significant changes in stiffness [27].

However, reliable measurement of muscle stiffness after intense exercise is of great importance to gain a comprehensive understanding of the changes in muscle tissue.

## 2. Materials and Methods

Study Design: The present study represents a pilot study in which data were collected in the period from 21 June to 28 June 2023. A specially equipped room in the physiotherapy department of the University Hospital Brandenburg an der Havel was used to conduct this study. The study design was submitted to and approved by the ethics committee of Medical School Brandenburg Theodor Fontane (protocol code: 105052023-BO-E, 25 September 2023).

A total of 16 subjects were included in this study. The subjects were carefully selected to ensure a representative sample for the study of muscle fatigue and its effects.

Inclusion and exclusion: All subjects who participated in this study were included, provided they had no lower extremity or trunk injuries requiring treatment in the past 24 months. Care was taken to ensure that participants were physically able to perform single-leg flexion or extension on the leg press without concomitant diseases that could hinder these movements or have a lasting effect on muscle tone. Examples of such concomitant diseases are neurological diseases or acute injuries to the lower extremities or trunk.

Furthermore, study participants were excluded if they had completed intensive leg training the previous day or had otherwise subjected their legs to high levels of stress. This was considered to ensure that the subjects did not show any immediate effects of previous training or increased muscle fatigue at the beginning of the study, which could influence the results of the investigation.

At the beginning of this study, various exercises were tried to maximize muscle fatigue in the legs. These included leg extensions, static leg presses at 90° flexion, and dynamic leg presses. For the dynamic leg press, both 50 kg and 30 kg were used as loads.

Since the test subjects had different training states and fitness levels, it was decided to select the single-leg variant of the dynamic leg press with 30 kg as the weight. This decision was also based on the goal of ensuring an even load and fatigue of the leg muscles for all participants.

In addition, it was possible to ensure that the participants could perform the exercise in a controlled and correct manner without overloading their joints or muscles.

This was particularly important to prevent injuries and to ensure the safety of the subjects during this study.

**Figure 1:**
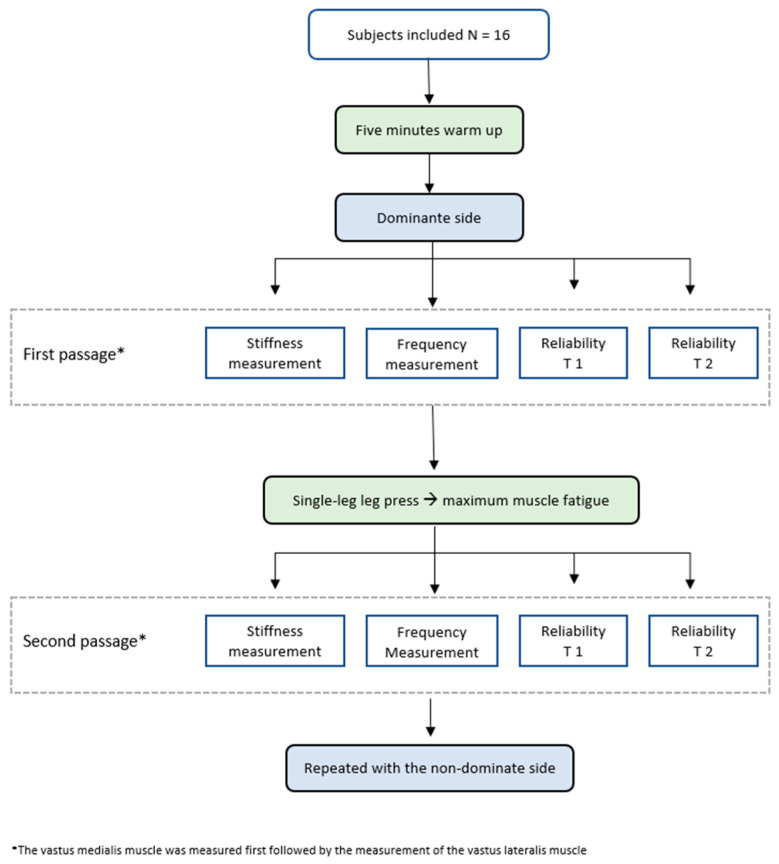
Study flow.

The experimental protocol (Figure 1) commenced with a preparatory phase utilizing the ERGO-FIT Cycle 400 ergometer, during which participants engaged in a five-minute warm-up at 100 watts and a cadence of 70 ± 5 revolutions per minute to activate and prime their leg muscles. Following this, participants assumed a recumbent position on a couch with a roller strategically positioned under the posterior aspect of the knee to induce maximal relaxation in the leg musculature. The initial data acquisition phase was executed sequentially by Tester 1 (T1) and Tester 2 (T2), focusing on obtaining values for muscle stiffness and muscle frequency.

**Figure 2:**
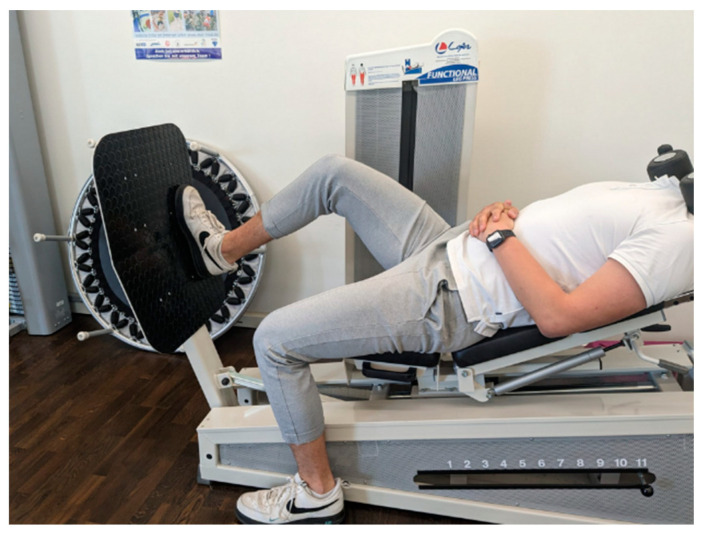
Single-leg leg press right.

Subsequently, participants assumed a seated position on the leg press apparatus, which was augmented with an additional 30 kg of weight. They then performed single-leg leg extensions with their dominant leg (Figure 2) until the point of maximal fatigue, defined as the subjective inability to execute further repetitions. Muscle stiffness and muscle frequency were subsequently assessed using the MyotonPro, with T1 and T2 conducting the measurements while the roller remained in place beneath the knee.

**Figure 3:**
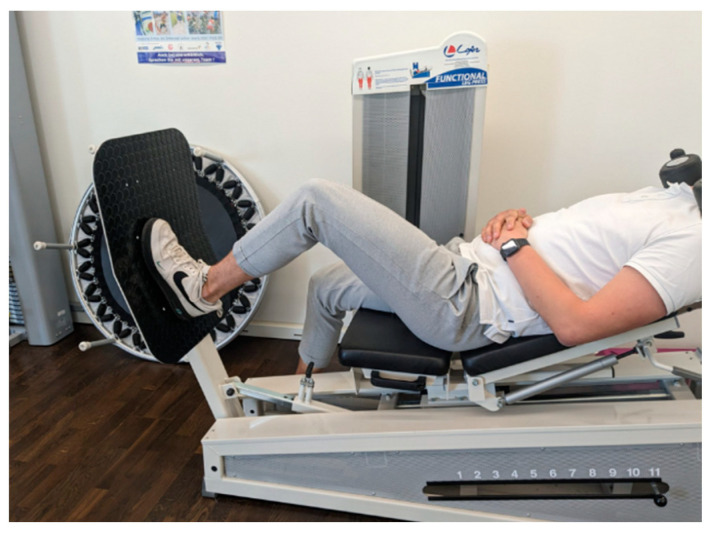
Single-leg leg press left.

**Figure 4:**
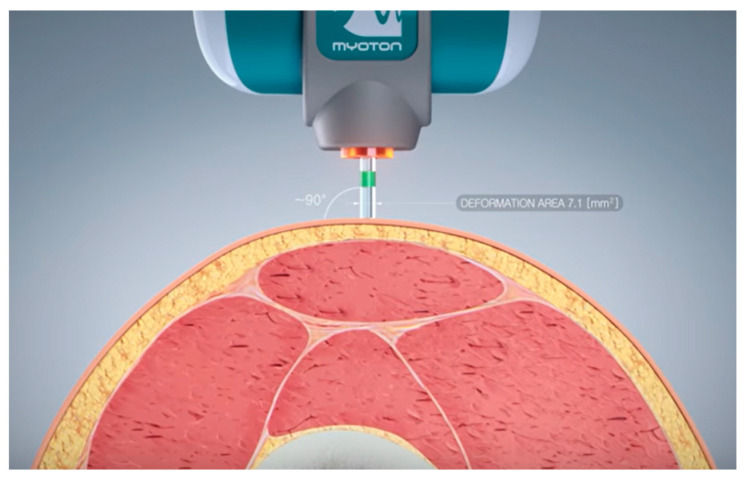
Handling the measurement of the MyotonPro [28].

This protocol was iterated on the non-dominant leg (Figure 3). Two independent testers were incorporated into this study to assess reliability. Prior to and subsequent to inducing maximal muscle fatigue in the dominant leg, both testers measured the vastus medialis and vastus lateralis. The MyotonPro was consistently maintained at a 90° angle (Figure 4) to the anatomical axis of the test subject to ensure uniformity. A visual marker, denoted by a cross on the skin, was utilized to demarcate the precise measurement point and persisted visibly post-exercise, confirming the stability of the measurement reference.

This meticulous approach to marking the muscle point with a cross served to standardize the orientation for both testers and mitigate potential sources of error stemming from variations in MyotonPro placement. This methodological refinement contributes to heightened measurement reliability and bolstered precision in result interpretation. The outlined procedures were subsequently replicated on the non-dominant leg, adhering rigorously to the established protocol.

Devices used: A range of devices were employed in the execution of this study. The assessment of muscle stiffness and muscle Frequency in the leg muscles was conducted utilizing the MyotonPro. The Lojer leg press apparatus was instrumental in regulating the load on the leg muscles and facilitating controlled leg extensions. To initiate the warm-up of the participants’ muscles, the ERGO-FIT Ergometer Cycle 400 was employed.

Statistical analysis: The present study undertook a comprehensive statistical analysis employing various software programs. Data acquisition utilized the MyotonPro and the Myoton Application for Windows, with the exported data undergoing sorting and checks for logical consistency in Microsoft Excel. Identified errors or outliers were rectified to ensure the data’s appropriateness for subsequent analyses. Subsequently, the Statistical Package for the Social Sciences (SPSS V29.0) was employed for the statistical analysis.

Internal consistency measures were applied to assess the reliability of the gathered measurements, with Cronbach’s alpha serving as a tool to evaluate the data’s consistency and accuracy. The analysis focused on various aspects of the collected data, ensuring their reliability and statistical significance.

The findings of this study were reported comprehensively, providing detailed information on the statistical significance and reliability of the collected data. It is noteworthy that due to the limited size of the cohort, no significant *p*-values could be attained. The cohort size proved to be a limiting factor, constraining the generalizability of the results. Quality control measures, such as checks for logical consistency and error corrections, were implemented throughout the process to ensure the overall quality of the data.

## 3. Results

### 3.1. Demographic Data

A total of 16 participants were recruited for this study, comprising seven males and nine females. The mean age of the participants was 25 years, with a standard deviation of ±3.46 years. Anthropometric measures indicated an average height of 170.8 cm ± 10.2 cm and a weight of 70 kg ± 11.3 kg, resulting in a mean body mass index (BMI) of 23.82, with a standard deviation of ±2.47.

In terms of handedness, only two participants identified as left-handed, while the remaining 14 were right-handed. The participants achieved an average of 140 repetitions, with a standard deviation of ±60 repetitions. The average execution time for the task under a 30 kg load was 3 min and 45 s, with a standard deviation of ±1 min and 15 s.

### 3.2. Dominant Side

#### 3.2.1. Stiffness Evaluation

**Figure 5:**
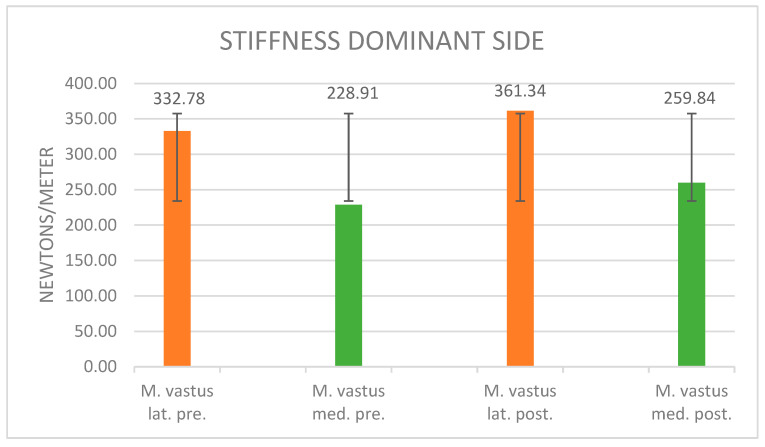
Stiffness values of the dominant side before and after muscle fatigue and the standard deviation.

Figure 5 displays the muscle stiffness, measured in newtons per meter (N/m), on the y-axis, ranging from 0 N/m to 400 N/m. The x-axis enumerates four distinct measurements. Initially, assessments of the vastus lateralis (orange) and vastus medialis (green) muscles are conducted in a resting state. Subsequently, measurements of the same muscles are taken following the induction of maximal muscle fatigue.

Upon maximal fatigue, the muscle stiffness of the dominant side of the vastus lateralis muscle exhibited an increase of 8.6%, ascending from 332.78 N/m to 361.34 N/m. Similarly, the muscle stiffness of the vastus medialis increased by 13.5% after maximal muscle fatigue, elevating from 228.91 N/m to 259.84 N/m. The corresponding standard deviations are reported as 71.56 (vastus lat. pre.), 45.8 (vastus med. pre.), 80.86 (vastus lat. post.), and 63.66 (vastus med. post.).

#### 3.2.2. Frequency Evaluation

**Figure 6:**
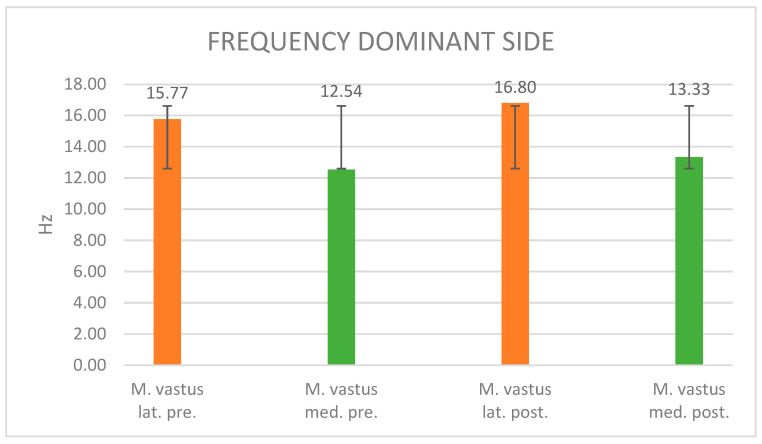
Frequency values of the dominant side before and after muscle fatigue and the standard deviation.

The description of the figure is now omitted as it does not differ from the description of Figure 5. Following maximal fatigue (Figure 6), the muscle frequency of the dominant side of the vastus lateralis exhibited a discernible 6.5% augmentation, increasing from an initial 15.77 Hz to a post-fatigue 16.8 Hz. Similarly, the muscle frequency of the vastus medialis displayed a 6.3% increase after maximal muscle fatigue, rising from an initial 12.54 Hz to a post-fatigue 13.33 Hz. The respective standard deviations are reported as 2.65 (vastus lat. pre.), 1.04 (vastus med. pre.), 3.23 (vastus lat. post.), and 1.52 (vastus med. post.).

### 3.3. Non-Dominant Side

#### 3.3.1. Stiffness Evaluation

**Figure 7:**
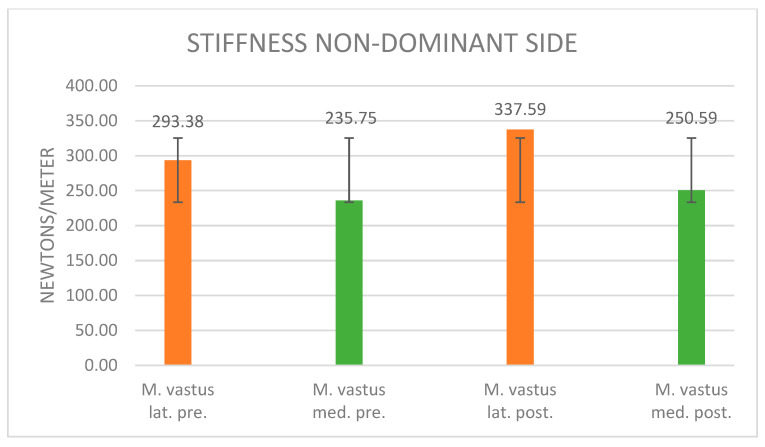
Stiffness values of the non-dominant side before and after muscle fatigue and the standard deviation.

The description of the figure is now omitted as it does not differ from the description of Figure 5. Figure 7 illustrates the alteration in muscle stiffness on the non-dominant side for the vastus lateralis and vastus medialis muscles following maximal fatigue. The muscle stiffness of the non-dominant vastus lateralis increased notably by 15.1%, surging from an initial 293.38 N/m to a post-fatigue level of 337.59 N/m. Correspondingly, the muscle stiffness of the vastus medialis experienced a 6.3% increment post-maximal muscle fatigue, elevating from 235.75 N/m to 250.59 N/m. The associated standard deviations are recorded as 68.19 (vastus lat. pre.), 43.38 (vastus med. pre.), 66.58 (vastus lat. post.), and 68 (vastus med. post.).

#### 3.3.2. Frequency Evaluation

**Figure 8:**
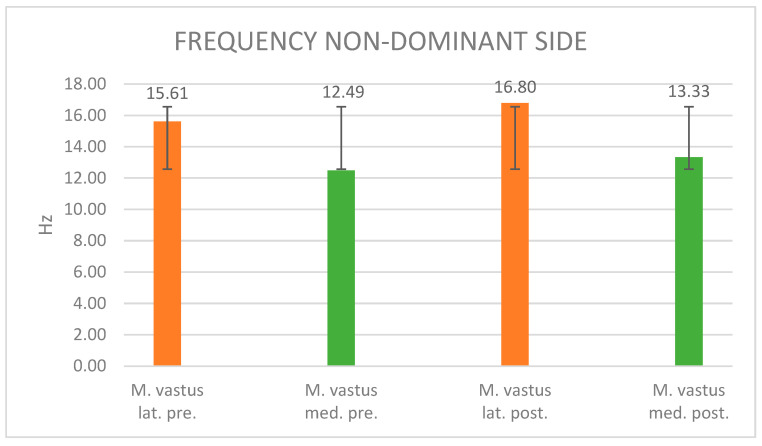
Frequency values of the non-dominant side before and after muscle fatigue and the standard deviation.

The description of the figure is now omitted as it does not differ from the description of Figure 5. Figure 8 delineates the alteration in muscle frequency on the non-dominant side, specifically for the vastus lateralis and vastus medialis muscles following maximal fatigue. The muscle frequency of the non-dominant vastus lateralis exhibited a discernible 7.6% increase, rising from an initial 15.61 Hz to a post-fatigue 16.8 Hz. Similarly, the muscle frequency of the vastus medialis displayed a 6.7% increment post-maximal muscle fatigue, ascending from an initial 12.49 Hz to 13.33 Hz. The associated standard deviations are reported as 2.69 (vastus lat. pre.), 1.01 (vastus med. pre.), 3.23 (vastus lat. post.), and 1.52 (vastus med. post.).

### 3.4. Reliability

Table 1 provides a comprehensive overview of the alterations in Cronbach’s alpha coefficients observed during the assessment of the vastus lateralis and medialis muscles by Tester 1 and Tester 2, both pre- and post-maximal muscle fatigue. Each assessment comprised eight items.

In the evaluation of the right vastus lateralis and medialis muscles by Tester 1, a Cronbach’s alpha coefficient of 0.75 was attained. Conversely, when the same measurements were conducted by Tester 2, the Cronbach’s alpha coefficient diminished to 0.49.

On the left side, the assessment by Tester 1 yielded a Cronbach’s alpha coefficient of 0.88 for the vastus lateralis and medialis muscles. However, when the assessments were carried out by Tester 2, the Cronbach’s alpha coefficient declined to 0.6.

Table 2 elucidates the fluctuations in Cronbach’s alpha coefficients when both Tester 1 and Tester 2 conduct assessments on the vastus lateralis muscle before and after maximal muscle fatigue, as well as the vastus medialis muscle before and after maximal muscle fatigue. A set of 8 items was incorporated for each analysis.

The outcomes reveal a Cronbach’s alpha coefficient of 0.8 for the vastus lateralis muscle, while the vastus medialis muscle demonstrates a Cronbach’s alpha coefficient of 0.83.

Table 3 delineates the modifications in Cronbach’s alpha coefficients when both Tester 1 and Tester 2 jointly conduct assessments on the vastus lateralis muscle before and after maximal muscle fatigue, as well as the vastus medialis muscle before and after maximal muscle fatigue. Each analysis incorporated eight items for the respective muscle groups.

The outcomes demonstrate that Cronbach’s alpha coefficient for the vastus lateralis muscle is 0.72 before maximal muscle fatigue and 0.74 after fatigue. Similar patterns were observed for the vastus medialis muscle, with Cronbach’s alpha coefficient being 0.79 before maximal muscle fatigue and 0.65 after fatigue.

## 4. Discussion

This research is significant as it investigates the evaluation of muscle stiffness and frequency through the utilization of the MyotonPro device, providing insights into its prospective clinical uses.

Demographic results: The findings pertain to a study with the participation of 16 subjects, comprising seven males and nine females. It is important to note that the gender distribution in this sample is not uniform, warranting caution in interpreting the study outcomes, and potential gender differences should be considered.

The mean age of the participants was 25 years, with a standard deviation of ±3.46 years, suggesting that the study population predominantly represents a relatively young age group. The average height of the subjects was 170.8 cm, with a standard deviation of ±10.2 cm. Subjects’ weight averaged 70 kg, with a standard deviation of ±11.3 kg. Calculating from these data, the average Body Mass Index (BMI) was 23.82, with a standard deviation of ±2.47. These results indicate that participants fall within the normal BMI range, and there is no extreme weight distribution in the study sample.

Participants’ dominant leg for categorization in this study was established through a combination of their explicit statements and the assessment of which leg they identified as their supporting limb. This comprehensive approach ensured a clear determination of the dominant leg in the research context.

The average number of repetitions performed was 140 ± 60, indicating variability in the repetitions among subjects, possibly attributed to individual differences in muscle fatigue and performance. The average execution time with a 30 kg load was 3 min and 45 s ± 1 min and 15 s. These results offer insights into the duration of the exercise performed by subjects, with observed variability in measured times hinting at individual differences in performance and endurance among participants.

Stiffness: The current findings reveal alterations in muscle stiffness both before and after maximal muscle fatigue, occurring on both the dominant and non-dominant sides of the vastus lateralis and medialis muscles. On the dominant side, there was an 8.6% increase in muscle stiffness in the vastus lateralis and a 13.5% increase in the vastus medialis. Conversely, on the non-dominant side, the changes were more pronounced, with a 15.1% increase in muscle stiffness observed in the vastus lateralis and a 6.3% increase in the vastus medialis.

These results indicate that muscle fatigue induces heightened muscle stiffness, manifesting differently across various muscles and sides. It appears that on the dominant side, the emphasis is more on the vastus medialis muscle, while on the non-dominant side, the vastus lateralis muscle is subjected to more stress. This discrepancy could be attributed to individual differences in muscle activation and control.

Additionally, it is noteworthy that the baseline stiffness of the vastus lateralis muscle is higher on both sides, potentially contributing to the observed differences [29,30]. However, it is crucial to acknowledge that these interpretations are preliminary, and further investigations are necessary to explore the exact mechanisms and relationships among muscle stiffness, muscle activation, and muscle fatigue.

Frequency: The current results suggest alterations in the muscle frequency of both vastus lateralis and medialis muscles before and after maximal muscle fatigue on both the dominant and non-dominant sides. On the dominant side, a 6.5% increase in muscle frequency was noted in the vastus lateralis, and a 6.3% increase was observed in the vastus medialis. Conversely, on the non-dominant side, more substantial changes were observed, with a 7.6% increase in muscle frequency in the vastus lateralis and a 6.7% increase in the vastus medialis.

Interestingly, the findings indicate a higher percentage increase in muscle frequency on the non-dominant side compared to the dominant side. This observation suggests that the non-dominant side may exhibit greater responsiveness to loading and fatigue, potentially employing an increased muscle frequency as a compensatory mechanism [23,25].

Maximal muscle fatigue induces heightened muscle stiffness and muscle frequency, as observed in both dominant and non-dominant legs. This insight holds pivotal significance for rehabilitation and sports performance, indicating that fatigue elicits distinct effects on specific muscle groups [8]. Rehabilitation programs and training protocols may necessitate tailored considerations for these distinctions to augment recovery and optimize performance [31]. The findings contribute invaluable insights into the impact of fatigue on muscle biomechanics, particularly regarding muscle stiffness and frequency. Future research endeavors and clinical practices can build upon these findings to formulate targeted interventions, enhance measurement reliability, and address inherent limitations for a more comprehensive comprehension of muscle fatigue.

This study’s revelations have the potential to inform the development of precise interventions and training protocols by illuminating discernible alterations in muscle stiffness and frequency. Customizing rehabilitation and training regimens based on these findings has the potential to ameliorate muscle function, optimize performance, and mitigate injuries in both athletes and rehabilitation patients [32]. However, a note of caution is warranted due to this study’s limited and potentially biased sample size, particularly concerning gender differences. Subsequent research could extend these findings by delving into the specific physiological mechanisms underlying changes in muscle stiffness and frequency.

Further exploration of the enduring effects of fatigue and the inclusion of a more diverse sample size would enrich the understanding of the intricate relationship between muscle stiffness, fatigue, and performance optimization. While this study alludes to alterations in muscle stiffness and frequency, specific physiological mechanisms necessitate in-depth investigation. Grasping these mechanisms can furnish insights into the nexus between muscle fatigue, recovery, and the observed changes, guiding the development of more precise interventions for rehabilitation and sports performance.

It has been observed that on the dominant side, the Vastus Medialis muscle experiences a higher increase in stiffness compared to the Vastus Lateralis. Conversely, on the non-dominant side, the pattern is reversed, with the Vastus Lateralis exhibiting higher stiffness values compared to the Medialis. This insight suggests that during maximal muscle fatigue on the dominant side, the Vastus Medialis undergoes a greater increase in stiffness, thereby potentially elevating its risk of injury [8]. On the non-dominant side, however, it is the Vastus Lateralis that is affected.

Reliability: In evaluating Cronbach’s alpha values, the following classification was employed: <0.5 deemed unacceptable, >0.5 considered poor, >0.6 questionable, >0.7 acceptable, >0.8 good, and >0.9 very good. The internal consistency, reflected by Cronbach’s alpha, exhibited variability across testers and muscle groups. For the right vastus lateralis and medialis muscles, Tester 1 achieved an acceptable Cronbach’s alpha of 0.75, while Tester 2’s value was deemed unacceptable at 0.49. On the left side, Tester 1 demonstrated a good Cronbach’s alpha of 0.88, whereas Tester 2’s value was questionable at 0.6. Diminished Cronbach’s alpha values could be attributed to incorrect apparatus manipulation or subject motion [25,26,33,34,35].

The present study reveals Cronbach’s alpha values of 0.8 for the vastus lateralis muscle and 0.83 for the vastus medialis muscle. These values signify good internal consistency between measurements of both muscle groups, indicating reliable results. While these values are below those of other studies, such as Bizzi et al. [26], it is crucial to note that in the cited study, the vastus lateralis muscle was deemed unacceptably measurable, unlike in the present study. This suggests that the methodology and measurement choices in the current study were reliable, yielding dependable results.

Cronbach’s alpha values for the vastus lateralis muscle were 0.72 and 0.74 before and after maximal muscle fatigue, respectively. Similar results were obtained for the vastus medialis muscle, with Cronbach’s alpha at an acceptable 0.79 before fatigue and a questionable 0.65 after fatigue. These outcomes indicate acceptable internal consistency in muscle stiffness measurements before and after maximal muscle fatigue, providing reliable and reproducible information essential for the validity of the measurement method used.

Comparable findings were reported by Chang-Young et al., where good to very good reliability values were achieved [34]. This further supports the validity of the present results and underscores the reliability of muscle stiffness measurements as a method for assessing muscle fatigue.

For future studies, it is imperative that the testers familiarize themselves with the MyotonPro and possess a thorough understanding of its technology. Proper operation of the MyotonPro involves achieving an optimal 90% alignment with the target tissue, and it is crucial not to rotate the device. Incorrect handling may result in inaccurate, either elevated or diminished, values being recorded by the MyotonPro. Additionally, maintaining the impulse interval, the frequency at which the measurement probe assesses the tissue, at 10 times per second or higher, is essential to minimize measurement outliers.

The inclusion of electromyography (EMG) for monitoring muscle activity should also be an integral part of this study. This incorporation aids in identifying arbitrary muscle activities and allows for the exclusion of such activities during resting measurements.

Limitations: The current study is subject to certain constraints that warrant consideration. Primarily, the participant pool is relatively small, with a sample size of n = 16, potentially limiting the generalizability of the findings. Enlarging the sample would enhance representativeness and bolster statistical power.

Moreover, there is a need for more precise test and measurement protocols to mitigate potential fluctuations in the recorded measurements. Another avenue for enhancing reliability involves the inclusion of a third tester, ensuring the verification of measurement consistency across different assessors.

Regrettably, due to time constraints, the reordering of subjects and additional testing were not feasible. However, these aspects could be addressed in subsequent studies to validate the results and explore potential long-term effects.

Additionally, five participants reported experiencing fatigue in the gluteus maximus muscle rather than the targeted vastus lateralis and medialis muscles during the leg press. This underscores the necessity of incorporating additional muscles like the rectus femoris and biceps femoris in future studies to present a more comprehensive understanding of the muscular response to fatigue.

In summary, these limitations are offered as considerations for future research endeavors aimed at refining methodology, expanding sample sizes, and incorporating more pertinent muscle groups. Such adjustments hold the promise of rendering results more precise and meaningful, thereby advancing our comprehension of muscle fatigue and its implications.

## 5. Conclusions

This study establishes the MyotonPro as a satisfactory measurement device for the evaluation of muscle stiffness. Notably, post-maximal muscle fatigue and augmentation in muscle stiffness were evident on both the dominant and non-dominant sides. Furthermore, an increase in muscle frequency was observed in the vastus lateralis and medialis muscles following maximal muscle fatigue. Nevertheless, for enhanced reliability, additional investigations are warranted to validate these findings and explore various facets of the muscular response to fatigue. These endeavors will contribute to refining the responsiveness of the MyotonPro, fortifying its utility in clinical applications.

## Figures and Tables

**Table 1:** Cronbach’s alpha values of vastus lateralis and medialis muscles of Tester 1 and 2, before and after maximal muscle fatigue. The right and left sides were considered separately.

	Cronbach’s Alpha Tester 1	Cronbach’s Alpha Tester 2	Number of Items
M. vastus lat. and med. right	0.75	0.49	8
M. vastus lat. and med. left	0.88	0.6	8

**Table 2:** Cronbach’s alpha values of vastus lateralis and medialis muscles of Tester 1 and 2, before and after maximal muscle fatigue. The right and left side.

	Cronbach’s Alpha	Number of Items
M. vastus lateralis (pre and post)	0.8	8
M. vastus medialis (pre and post)	0.83	8

**Table 3:** Cronbach’s alpha values of vastus lateralis and medialis muscles of Tester 1 and 2, right and left side. A distinction was made between before and after maximum muscle fatigue.

	Cronbach’s Alpha (Pre)	Cronbach’s Alpha (Post)	Number of Items
M. vastus lat.	0.72	0.74	8
M. vastus med.	0.79	0.65	8

## Data Availability

Additional data are available after a request from the corresponding authors.
